# Determination of the Use of* Lactobacillus plantarum* and* Propionibacterium freudenreichii* Application on Fermentation Profile and Chemical Composition of Corn Silage

**DOI:** 10.1155/2017/2038062

**Published:** 2017-04-19

**Authors:** Norafizah Abdul Rahman, Mohd Ridzwan Abd Halim, Noraniza Mahawi, Hazira Hasnudin, Jameel R. Al-Obaidi, Norhani Abdullah

**Affiliations:** ^1^Agro-Biotechnology Institute Malaysia (ABI), c/o MARDI Headquarters, 43400 Serdang, Selangor, Malaysia; ^2^Crop Science Department, Faculty of Agriculture, Universiti Putra Malaysia (UPM), 43400 Serdang, Selangor, Malaysia; ^3^Institute of Tropical Agriculture, Universiti Putra Malaysia (UPM), 43400 Serdang, Selangor, Malaysia

## Abstract

Corn was inoculated with* Lactobacillus plantarum* and* Propionibacterium freudenreichii* subsp.* shermanii* either independently or as a mixture at ensiling, in order to determine the effect of bacterial additives on corn silage quality. Grain corn was harvested at 32–37% of dry matter and ensiled in a 4 L laboratory silo. Forage was treated as follows: bacterial types: B0 (without bacteria-control), B1* (L. plantarum)*, B2 (*P. freudenreichii* subsp.* shermanii*), and B3 (combination of* L. plantarum* and* P. freudenreichii* subsp.* shermanii*). Each 2 kg of chopped forage was treated with 10 mL of bacterial culture and allowed to ferment for 27 days. The first experiment determined the most suitable wavelength for detection of bacteria (490 nm and 419 nm for B1 and B2, resp.) and the preferable inoculation size (1 × 10^5^ cfu/g). The second experiment analysed the effect of B1 and B2 applied singly or as a mixture on the fermentation characteristics and quality of corn silage.* L. plantarum* alone increased crude protein (CP) and reduced pH rapidly. In a mixture with* P. freudenreichii*, the final pH was the lowest compared to other treatments. As a mixture, inclusion of bacteria resulted in silage with lower digestibility than control. Corn silage treated with* L. plantarum* or* P. freudenreichii* either alone or mixed together produced desirable silage properties; however, this was not significantly better than untreated silage.

## 1. Introduction

The ability of corn silage additives to conserve the nutritive value of a crop during fermentation is very important. The preservation of forage crops as silage depends on the production of sufficient acid to inhibit the activity of undesirable microorganisms under anaerobic conditions. Bacteria inoculated during ensiling dominate epiphytic bacteria in the forage and enable successful reduction of the pH, thus reducing losses in yield and nutritive value associated with silage production [1]. Additives are used to prevent or reduce the growth of undesirable microorganisms such as clostridium bacteria and fungus in silages, thus enhancing silage fermentation [2].

Homofermentative and heterofermentative inoculants can improve fermentation quality and the stability of silage through the production of large amounts of organic acid that maintains forage in acidic condition [3, 4]. Some studies have shown that adding only homofermentative bacteria improves fermentation quality (although the silage is susceptible to aerobic deterioration at feed out), while heterofermentative bacteria are more likely to prolong aerobic stability upon air exposure [5, 6].

Due to the lack of good quality feed and limited grazing area for ruminants in Malaysia, intensive research to look for alternatives and substitutes is required, in order to reduce the burden of feed imports by increasing the use of indigenous feed resources. Appropriate strategies to enhance nutrient quality for improved rumen function and to manage supplements are of importance to ruminant feeding. The use of microbial additives for silage production is not commonly practised in Malaysia, as evidence for its efficacy in climates such as those seen in Malaysia is lacking. Therefore, information on the benefits and effectiveness of microbial additives in fermentation of silage in tropical environments such as Malaysia is required, in order to make appropriate recommendations for silage production under local conditions.

To ensure sustainable ruminant production, the availability of feed throughout the year is crucial. Hence, certain strategies in feed production should be developed. To this effect, fodder conservation through appropriate ensiling is required [7, 8]. Due to the high humidity in tropical countries such as Malaysia, plant materials like corn can deteriorate easily [8]. Therefore, the aim of this study was to determine the effects of selected bacterial species (both hetero- and homofermentative) as additives in corn silage fermentation in a tropical environment.

The successful preservation of forage crops as silage depends on the production of sufficient organic acids to inhibit the activity of undesirable microorganisms under anaerobic conditions. According to [9], using* L. plantarum* as additives has resulted in the enhancement of the lactic acid fermentation compared to control (no bacterial additives). Even though the inoculant application rate was similar to the epiphytic LAB population, adding bacteria increased the aerobic stability, increased acetic acid production, and reduced the ammonia-N concentration [2]. Nevertheless, a review reported by [10], on 19 studies conducted at Kansas State University involving inoculated corn silage, had 1.3% higher DM recoveries, 1.8% more efficient gains of beef cattle, and 1.6 kg more gain per tonne of crop ensiled. Compared to untreated silage, inoculation using* L. plantarum* was shown to improve the fermentation quality in recent study [11].

Studies by [12] showed that culture mixed with* P. freudenreichii* was highly effective against spoilage yeasts. Furthermore, silage treated with inoculant containing* P. freudenreichii* tended to reduce mould counts and improved aerobic stability of the silage [13]. Microbial inoculants containing only homolactic bacteria or those containing homolactic and heterolactic bacteria can be used to improve the fermentation and aerobic stability of bermudagrass haylage [13]. In general,* Propionibacterium* have been effective in situations where the decline in pH is slow and (or) when the final pH of silage has been relatively high (>4.2 to 4.5).

Although literature summaries are encouraging, caution should be used when interpreting such data because all inoculants are not equal and the conditions (e.g., rate of application, inoculant viability, bacteria strain used as additives, crop, environmental temperature, wilting period, and moisture levels) varied markedly among the studies. As many have pointed out in the past, products with organisms with the same name are not necessarily the same organism and may not have the same effectiveness. The organism(s) from microbial inoculants must be present in sufficient numbers to effectively dominate the fermentation. Thus the most commonly recommended inoculation rate supplies 100,000 (or 1 × 10^5^) organisms per gram of wet forage. There is little evidence that suggests that doubling or tripling this amount (e.g., 200–300,000 cfu/g) is beneficial [4, 14].

As a consequence, the main objective of this research was to evaluate the effect of applying* L. plantarum* (homofermentative) and* P. freudenreichii* subsp.* shermanii* (heterofermentative) either alone or as a mixture on the fermentation quality of corn silage, and therefore to determine the most suitable choice of bacterial additives to be used for corn silage production.

## 2. Materials and Methods

### 2.1. Microbial Preparation

Two bacterial species* Lactobacillus Plantarum* ATCC® 8014™* (L. plantarum)* and* Propionibacterium freudenreichii* subsp.* shermanii* (ATCC 13673™)* (P. freudenreichii)* were used in this study. Both cultures were purchased from the American Type Culture Collection (ATCC). The freeze-dried powder of* Lactobacillus plantarum* was mixed with 1.0 mL of De Man, Rogosa, Sharp (MRS) broth. The contents were transferred aseptically into a 15 mL universal bottle. Several drops of the culture were used to inoculate both MRS broth and MRS agar plates, which were then both incubated anaerobically (37°C, 48 h).* Propionibacterium freudenreichii* subsp.* Shermanii* was mixed with 1.0 mL of nutrient broth (NB) and transferred aseptically into a 15 mL universal bottle. Several drops of the culture were inoculated on Reinforced Clostridial medium (Oxoid CM149) agar slant and plates. The cultures were incubated anaerobically (30°C, 48–72 h).

### 2.2. Determination of Optimal Wavelength for Bacterial Optical Density Measurements

The optical densities of 3 mL of bacterial solutions were determined by using a UV-VIS Spectrophotometer daily at the same time after inoculation.* L. plantarum* was measured at four different wavelengths (490, 520, 545, and 600 nm), while* P. freudenreichii* was analysed at 419, 578, 650, and 750 nm. The wavelengths selected were based on several reports [15–18] for determining the absorbance of these bacterial cultures. Observations were recorded at the same time after inoculation, from day 1 to day 4* (L. plantarum)* and day 1 to day 7* (P. freudenreichii)*.

### 2.3. Ensiling Corn with Bacterial Additives

A two-factor experiment was conducted to evaluate the effects of bacterial inoculation size on corn silage fermentation. The two factors were as follows: bacterial type: B0 (without bacteria, control), B1* (L. plantarum)*, B2 (*P. freudenreichii* subsp.* shermanii*), and B3 (combination of* L. plantarum* and* P. freudenreichii *subsp.* shermanii*) and inoculation size: T0 (without bacteria, control), T1 (1 × 10^5^ cfu/kg), a typical inoculation size [1, 4, 19], T2 (2 × 10^5^ cfu/kg), double this size, and T3 (0.5 × 10^5^ cfu/kg), half the size of a typical inoculation.

Corn (Suwan 3) was grown on an experimental plot at the Department of Crop Science, Faculty of Agriculture, University Putra Malaysia (UPM) (2.9917°N, 101.7163°E), Malaysia. It was grown in 85.4 m × 68.0 m planting areas with 5 rows of each plot and 50 holes per row. Whole crop grain corn was harvested at 35% DM during dough maturity stages, cut at 5–10 cm above ground, and later chopped at approximately 3–8 cm length by using a forage chopper.

Two kilograms of chopped materials was placed on polyethylene sheet and sprayed with 10 mL of prepared bacterial solution. B1, B2, and B3 were sprayed at four different inoculation sizes. After 27 days of ensiling, 400 g samples were collected randomly from the middle part of the silo for chemical analysis, in order to determine the quality and fermentation products of the corn silage.

### 2.4. Chemical Analysis

Samples were collected, dried in a forced-air oven (60°C, 48 h), and ground to pass a 1 mm sieve using an industrial cutting mill (RETSCH, Germany). The water soluble carbohydrate (WSC) concentration was determined using the method described by [20], and concentrations of neutral detergent fibre (NDF) and acid detergent fibre (ADF) were measured using the FibreCap™ 2021/2023 [21], Fibre Analysis System by FOSS (FOSS, Denmark). The concentrations of DM (method 934.01), CP (method 984.13), and ADF (method 973.18) were analysed as described by the Association of Official Analytical Chemists [22]. Twenty grams of each silage sample was blended with 200 mL of deionized water and filtered with cheese cloth. The filtrated sample was used for determination of pH using an electronic digital pH meter (Mettler Toledo).

Organic acid was determined by using a gas chromatography (GC) mass spectrometry triple quadruple (QQQ) system (Scion TQ, Bruker). The autoinjector was set at a 1 : 10 split ratio and evaporated at 250°C. Analysis was conducted by using a DB-FFAP column (Agilent) (30 m × 250 um × 0.25 um size) with helium gas as the carrier at 1 mL/min. The oven was set to start at 50°C, hold for 2 minutes, increase to 240°C at the 20°C/min, and hold for 5 minutes, with a total run time of 16.5 minutes for each analysis. The results were analysed using MSWS 8.0 software to quantify the compounds.

Nonvolatile organic acid was extracted by using the method of [21], with modifications. For nonvolatile organic acid (NVOA), 50 g of fresh silage sample was blended with 100 mL of deionized water. The extract was filtered using cheese cloth and centrifuged (3,500 rpm, 4°C, 15 mins). Three mL of supernatant was added to 0.6 mL of 24% metaphosphoric acid in deionized water, and incubated overnight to inhibit bacterial activity in the samples. The sample was again centrifuged, and 0.5 mL of supernatant was taken for GC sample preparation. The derivatization of nonvolatile compounds to methyl ester was performed. One mL of 20% boron trifluoride (BF_3_) and 0.5 mL of 20 mM fumaric acid (as an internal standard) were added to 0.5 mL of supernatant and mixed well using a vortex mixer. The mixture was covered with aluminium foil and incubated at 37°C in a water bath overnight. 0.5 mL of chloroform was then added to the mixture and mixed well. The solution was left for 10 minutes to let it separate into two layers. The bottom solution was injected into the GC (0.2 uL per injection).

Volatile organic acid was extracted using the method of [21], with modifications. One hundred mL of deionized water was added to 50 g of silage and blended (medium speed, 1 min) using a commercial blender (Waring, USA). The extract was filtered using cheesecloth and centrifuged (4,000 ×g, 4°C, 15 mins). Three mL of supernatant was added to 0.6 mL of 24% metaphosphoric acid in deionized water and incubated overnight to inhibit bacterial activity in the samples. The sample was again centrifuged at the same speed and time, and 0.2 uL of supernatant was then injected into the GC.

### 2.5. Statistical Analysis

All results were subjected to analysis of variance (ANOVA) using a completely randomized design with four replications for all treatments. The data were further analysed using JMP version 10.0 (SAS Institute, Cary, NC, USA) statistical software. The differences between means were tested at a significance value of *p* < 0.05.

## 3. Results

### 3.1. Wavelength for Optimal Optical Density Measurements

**Figure 1:**
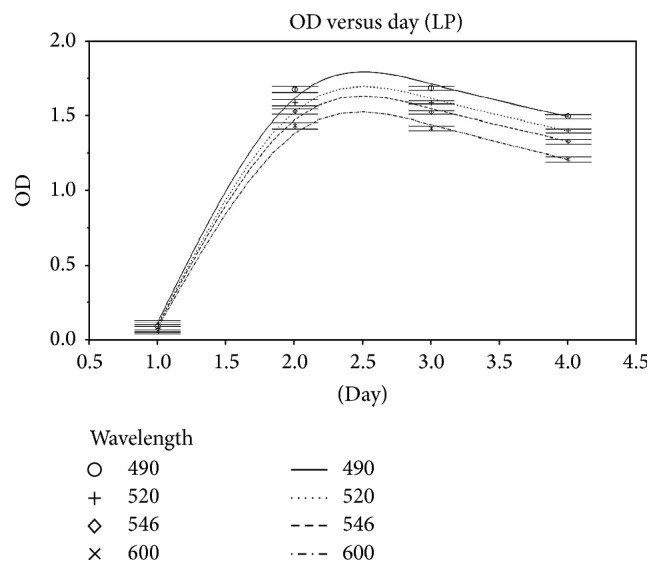
OD readings for* L. plantarum* at different wavelengths from days 1 to 4. The highest absorption was at 490 nm on day 2.

**Figure 2:**
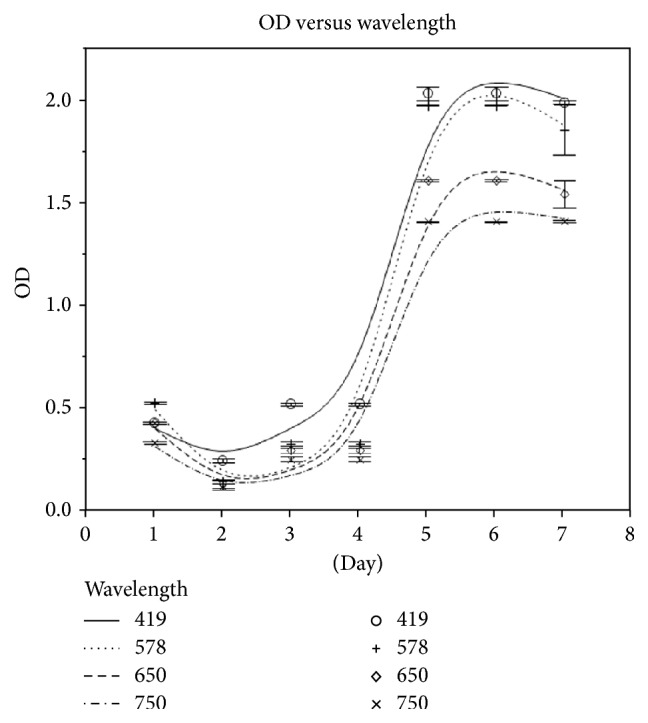
OD readings for* P. freudenreichii* at different wavelengths from days 1 to day 7. The highest OD reading was at 419 nm on day 5.

The OD reading for* L. plantarum* was highest at day 2 at 490 nm (Figure 1) and remained at the same level on day 3 (48 h). OD readings remained higher at 490 nm than other selected wavelengths. For* P. freudenreichii*, OD readings began to increase on day 5 and remained high until day 7 (Figure 2). 419 nm gave the highest OD readings for all observations.

### 3.2. Effect of Bacterial Additives and Inoculation Size on Silage Characteristics

Bacterial treatments affected the pH of silage, with the pH being significantly lower than control (*p* < 0.05). The mixture of both bacteria gave the lowest pH value (3.31). There was no significant difference in pH produced by different inoculation sizes (Table 1). Water soluble carbohydrate (WSC) measurements showed that only B3 gave a significantly lower WSC concentration (*p* < 0.05) compared to control (Table 1). Treatments with B1 and B2 were not significantly different from control (B0). Inoculation size at double (T2) and half (T3) resulted in no difference in WSC concentration compared to normal inoculation size (T1). Bacterial treatments did not affect lactic acid content in the silage compared to uninoculated silage (B0). Applying bacteria at normal (T1), double (T2), and half (T3) inoculation sizes produced a significant difference in pH but no significant difference at *p* < 0.05 for WSC and lactic acid concentration compared to control (T0).

### 3.3. Chemical Composition

The effects of bacterial treatments on pH, DM, CP, ADF, NDF, and WSC are shown in Table 2. Among all treatments, B3 produced the lowest pH (3.33), while the highest pH resulted from B2 (3.45). Treatment with* L. plantarum* (B1) and* P. freudenreichii* (B2) individually did not have a significant effect on silage pH, as the final pH was not different from control (B0). However, by applying a mixture of both bacteria, the pH reduction was significant compared to control (B0). Even though there was a significant difference at *p* < 0.01 among bacterial treatments, the final pH was considered low and acceptable for a good quality silage.

The CP, ADF, and NDF in the control silage were 7.51%, 31.08%, and 56.50%, respectively (Table 2). CP for silage treated with B1 (7.96%) differed significantly from control at *p* < 0.05.* L. plantarum* (B1) produced the highest CP among the other silages. ADF and NDF values were also significantly different and highest for B3 (35.47% and 63.11%, resp.), compared to control (31.08% and 56.50%, resp.) at *p* < 0.05. Bacteria in combination (B3) did not reduce fibre in silage, while other treatments were not significantly different compared to control (B0). The DM contents of all treatments ranged from 27.58% to 29.61%, showing no significant differences among treatments.

Water soluble carbohydrate concentration was significantly different for bacterial treatments at *p* < 0.01, compared to control. Combination bacteria (B3) showed the lowest WSC mean concentration (35.99 ug/mL) and were significantly different to control (52.71 ug/mL).

### 3.4. Organic Acid Composition

Lactic acid dominated organic acid production in the silage (Table 3). Acetic acid production was highest in the control (8.57 g/kg) and lowest for B1. The treatment with B1 produced high lactic acid and low acetic and propionic acids, suggesting that homolactic fermentation occurred in B1. Only propionic acid was significantly different among bacterial treatments, and B2 produced less propionic acid than control. Surprisingly, B2* (P. freudenreichii)* produced the least amount of propionic acid, suggesting that most of this acid was further converted to produce lactic and acetic acids. All treatments produced very little butyric acid due to the high levels of lactic acid production that prevented secondary fermentation.

## 4. Discussion

The success of producing silage of high quality depends on two main factors, firstly the nature of ensiling materials which determines the microbial population and secondly chemical composition while the second is the mechanism or strategy of pretreatment of the silage [23]. The use of inoculant prior ensiling is a common practice to hasten the fermentation process for ensuring good quality silage. Silages treated with adequate numbers of appropriate bacteria should be lower in pH, acetic acid, butyric acid, and ammonia-N but higher in lactic acid content. The microbial inoculants must be available in sufficient numbers to effectively dominate the fermentation [19].

The results showed that the optimum OD reading was obtained at 490 nm for* L. plantarum*. The absorbance started to increase on day 2 and remained the same on day 3, indicating that the optimum growth duration for* L. plantarum* takes place within 2 days of incubation [4, 17, 24].* P. freudenreichii* is an aerotolerant organism which cannot use oxygen for growth but can tolerate its presence. It grew well at 30°C under anaerobic conditions and formed creamy colonies in 5-6 days. For* P. freudenreichii*, 419 nm gave the highest OD readings. Good growth was detected by day 5. Other researchers have suggested a variety of optimal wavelengths for this species, such as 750, 720, 600, and 419 nm [25–27], with the choice of wavelength depending on the growth media used.

Bacterial treatments affected final silage pH values significantly (*p* < 0.05). Using bacteria inoculated either individually or in combination resulted in a lower final pH compared to control. The combination (B3) produced the lowest pH at the end of the fermentation. However, the final pH produced in the control (3.58) is considered acceptable. The inoculation size had no significant effect on pH and fermentation characteristics (water soluble carbohydrates and lactic acid) when the bacterial cultures (B1 (LAB) or B2 (PAB)) were inoculated individually. However, when the cultures were applied in combination, the low pH value indicated synergistic growth effects between the two bacterial species. Nevertheless, all treatments produced acceptable pH values (below pH 4.5). Such values are considered good as the undesirable bacteria that are involved in silage deterioration mainly grow at a pH above 4.0, with a pH below 4.5 preventing the growth of yeast and other undesirable silage bacteria [28, 29].

When LAB and PAB were combined, they produced a lower WSC concentration but there was no significant difference in LA concentration compared to control. The low amount of WSC in treated silage compared to uninoculated silage may have been due to the conversion of WSC to other end products by the inoculated bacteria [30]. Some studies have shown that corn silage inoculated with* P. freudenreichii*, with or without LAB, does not show significant differences in chemical composition due to the presence of similar bacterial species in the uninoculated silage [31].

The inoculation size should be sufficient to effectively dominate the fermentation. The most common inoculation size recommended for* L. plantarum* is 1 × 10^5^ cfu per gram of wet forage, and 10^5^ to 10^6^ cfu per gram of wet forage for other common silage bacteria. The inoculation size in this study had no significant effect on WSC and lactic acid. This result was in contradiction with findings by [32], who determined that a half inoculation size produced better fermentation quality and aerobic stability of the silage. The standard size (T1 (1 × 10^5^ cfu/g)) was the preferable inoculation rate for B1 and B2 either applied singly or in combination. Application of suitable bacterial inoculation size ensures better silage quality, prevents silage deterioration, and is also economical [4, 19].

Applying bacteria as a mixture (*L. plantarum *and* P. freudenreichii*) reduced the final pH significantly compared to control (without bacteria). However,* P. freudenreichii* applied individually resulted in higher final pH than other treatments. This was expected because* P. freudenreichii* is known to grow in a higher pH environment compared to* L. plantarum* [31, 33, 34].

*L. plantarum* increased CP production compared to other bacterial treatments, which may have been due to the higher production of protein in the form of nitrogen (N) content. Other bacterial treatments did not result in significantly different CP concentrations compared to control. However, the final DM and CP concentrations of the silage will depend on the type of forage used and the maturation time of this forage upon harvesting [35].

Fibre values (ADF and NDF) cannot be predicted by the availability of fermentation substrates. ADF and NDF are tightly associated with the amount of grain present in the grain to stover ratio (grain : stover). Corn forage generally has enough soluble sugars for completion of fermentation, regardless of ADF and NDF values. The ADF and NDF concentration are more relevant for grasses or alfalfa silage due to their higher fibre content compared to corn forage [36]. The mixture of* L. plantarum and P. freudenreichii* did not give a significant reduction in fibre, indicating that these bacteria have low fibrinolytic capabilities.

All treatments produced high lactic acid content, which was the highest among all organic acids. Treatment with* L. plantarum* produced high lactic acid (LA) and low acetic acid (AA) compared to other treatments. These results suggest that the fermentation was desirable, in that most of the sugar was converted to lactic acid rather than acetic acid, indicating that no secondary fermentation had occurred. Bacterial treatments produced a significant effect on water soluble carbohydrate and propionic acid production. Many studies have reported that lactobacilli are the dominant microbial population in forage crops and significantly contribute to silage fermentation [37, 38]. Water soluble carbohydrate (WSC) was highest in the* P. freudenreichii* treatment. Normal microflora existing in the control silage are likely to have consisted of a sizeable population of lactic acid bacteria, based on the high levels of lactic acid present in the control silage.

Previous studies have found that using homofermentative bacteria (such as* L. plantarum*) alone can lead to aerobic deterioration of silages, because these bacteria produce relatively increased levels of residual WSC and lactate, which are used as growth substrates by spoilage-causing yeasts and moulds [19, 39]. Other researchers have observed that inoculants containing propionic acid bacteria produce metabolites that benefit the growth of lactic acid bacteria [40]. In the present study, the addition of* P. freudenreichii* produced significantly high lactic acid and low propionic acid, suggesting that most of the sugar was converted to lactic acid rather than propionic acid. Applying* L. plantarum* either alone or in combination with* P. freudenreichii* produced a good silage in this study, but this was not a significant improvement compared to uninoculated silage.

## 5. Conclusion

This study concludes that inoculating silage with* L. plantarum* alone can increase CP and reduce pH rapidly. As a mixture with* P. freudenreichii*, the final pH was the lowest of all treatments. This mixture also caused significantly lower fibre digestibility compared to control. However, the nutritive value and fermentation quality of silages in this study were not significantly improved by inoculation at ensiling with* L. plantarum* or* P. freudenreichii* or using a combination of both, when compared to uninoculated silage. The silage treated with these selected bacteria either alone or as a mixture produced a similar quality as untreated silage.

## Figures and Tables

**Table 1:** Silage properties with different bacterial additives and inoculation sizes.

Treatments	pH	WSC	Lactic acid
*Bacteria (B)*			
B0	3.58^a^	56.11^a^	4445^a^
B1	3.39^b^	58.02^a^	4729^a^
B2	3.48^c^	49.62^ab^	5143^a^
B3	3.31^d^	40.23^b^	5239^a^
*p* > *F*	<0.0001	0.0002	0.6529

*Inoculation size (T)*			
T0	3.58^a^	14.32^a^	4445^a^
T1	3.39^b^	11.37^a^	5280^a^
T2	3.39^b^	17.98^a^	5199^a^
T3	3.40^b^	13.60^a^	4745^a^
*p* > *F*	0.0009	0.4503	0.6587

*Note*. B × T interaction was not significant. B0: no bacteria-control, B1: *L. plantarum*, B2: *P. freudenreichii *subsp*. Shermanii*, and B3: combination of *L. plantarum *and *P. freudenreichii *subsp*. Shermanii*; T0: control, T1: (1 × 10^5^ cfu/kg), T2: (2 × 10^5^ cfu/kg), and T3: (0.5 × 10^5^ cfu/kg); WSC: water soluble carbohydrate; a, b, c, and d: means in columns with similar letters were not significantly different (*p* > 0.05).

**Table 2:** Mean of pH, DM, CP, ADF, and NDF for bacterial treatment at day 27 of fermentation.

Nutrients						
BACT	pH	DM (%)	CP (%)	ADF (%)	NDF (%)	WSC (ug/ml)
B0	3.39^a^	27.58^a^	7.51^b^	31.08^b^	56.50^b^	52.71^ab^
B1	3.34^bc^	29.61^a^	7.96^a^	32.58^ab^	59.70^ab^	54.94^a^
B2	3.45^a^	28.87^a^	7.28^b^	33.53^ab^	60.34^ab^	43.49^bc^
B3	3.33^c^	27.84^a^	7.44^b^	35.47^a^	63.11^a^	35.99^c^
*p* > *F* (0.05)	<0.0001	0.1674	0.0143	0.0192	0.0309	<0.0001

*Note*. B0: no bacteria-control; B1: *L. plantarum*; B2: *P. freudenreichii *subsp.* Shermanii*; B3: combination of *L. plantarum* and *P. freudenreichii *subsp.* Shermanii*; BACT: bacteria; DM: dry matter; CP: crude protein; ADF: acid detergent fibre; NDF: neutral detergent fibre; WSC: water soluble carbohydrate; a, b, and c: means in columns with similar letters were not significantly different (*p* > 0.05).

**Table 3:** Amount of lactic acid (LA), acetic acid (AA), propionic acid (PA), and butyric acid (BA) (g/kg DM) for different bacterial treatments.

Bacteria	LA mean (g/kg)	AA mean (g/kg)	PA mean (g/kg)	BA mean (g/kg)
B0	26.96^a^	8.57^a^	0.54^a^	0.01^a^
B1	32.93^a^	2.94^b^	0.29^ab^	0.02^a^
B2	34.50^a^	7.29^a^	0.09^b^	0.01^a^
B3	23.89^a^	4.89^ab^	0.20^ab^	0.00^a^
*p* > *F*	0.091	0.0393	0.0202	0.1568

*Note*. B0: no bacteria-control; B1: *L. plantarum*; B2: *P. freudenreichii *subsp.* Shermanii*; B3: combination of *L. plantarum* and *P. freudenreichii *subsp.* Shermanii*. Differences shown by different letters for all rows for each column.

a, b: means in columns with similar letters were not significantly different (*p* > 0.05).
